# The Metabolic Reprogramming Evoked by Nitrosative Stress Triggers the Anaerobic Utilization of Citrate in *Pseudomonas fluorescens*

**DOI:** 10.1371/journal.pone.0028469

**Published:** 2011-12-01

**Authors:** Christopher Auger, Joseph Lemire, Dominic Cecchini, Adam Bignucolo, Vasu D. Appanna

**Affiliations:** Department of Chemistry and Biochemistry, Laurentian University, Sudbury, Ontario, Canada; Vrije Universiteit Brussel, Belgium

## Abstract

Nitrosative stress is an ongoing challenge that most organisms have to contend with. When nitric oxide (NO) that may be generated either exogenously or endogenously encounters reactive oxygen species (ROS), it produces a set of toxic moieties referred to as reactive nitrogen species (RNS). As these RNS can severely damage essential biomolecules, numerous organisms have evolved elaborate detoxification strategies to nullify RNS. However, the contribution of cellular metabolism in fending off nitrosative stress is poorly understood. Using a variety of functional proteomic and metabolomic analyses, we have identified how the soil microbe *Pseudomonas fluorescens* reprogrammed its metabolic networks to survive in an environment enriched by sodium nitroprusside (SNP), a generator of nitrosative stress. To combat the RNS-induced ineffective aconitase (ACN) and tricarboxylic acid (TCA) cycle, the microbe invoked the participation of citrate lyase (CL), phosphoenolpyruvate carboxylase (PEPC) and pyruvate phosphate dikinase (PPDK) to convert citrate, the sole source of carbon into pyruvate and ATP. These enzymes were not evident in the control conditions. This metabolic shift was coupled to the concomitant increase in the activities of such classical RNS detoxifiers as nitrate reductase (NR), nitrite reductase (NIR) and S-nitrosoglutathione reductase (GSNOR). Hence, metabolism may hold the clues to the survival of organisms subjected to nitrosative stress and may provide therapeutic cues against RNS-resistant microbes.

## Introduction

Nitric oxide (NO) is a gaseous free radical bestowed with several crucial roles in living organisms. It has emerged as an important endogenous signaling molecule in organisms as diverse as mammals and plants. It is usually derived from arginine with the aid of the enzyme nitric oxide synthase (NOS) and is known to be a modulator of blood pressure in mammals. NO also regulates information in nervous systems and is a messenger for mitochondrial functions [1]. In plants, the signaling roles of NO extend to germination, senescence, and cell wall construction [2]. NO is also synthesized in the phagocytes in response to microbial infection where in combination with ROS, it generates highly toxic derivatives that are utilized to combat the bacterial invasion [3], [4].

Nitrosative stress arises when the production of RNS outmatches an organism's ability to neutralize and dispose of them. These moieties are capable of damaging nucleic acids, lipids and amino acids. They disrupt proteins containing Fe-S clusters, transition metals, hemes, thiols and tyrosyl groups [5]. These RNS-triggered modifications inhibit essential cellular metabolism, lead to irreversible damage and eventually to the demise of the organism. Hence, it is not surprising that intricate strategies are elaborated by living systems to eliminate the dangers posed by RNS. Numerous heme proteins are known to be induced by nitrosative stress and have been shown to play a pivotal role in countering RNS. For instance, flavohemoglobin and cytochrome C reductase enable *E. coli* to be resistant to excessive NO [5], [6]. GSNOR, catalase and NAPDH-utilizing enzymes have all been reported to quell the toxic influence of RNS [4], [7]. Although the RNS-detoxifying role of these enzyme systems has been well documented, the participation of metabolism in combating the dangers posed by NO has yet to be fully uncovered.

In this study, we have evaluated the metabolic responses of *P. fluorescens* to nitrosative stress. Owing to its nutritional versatility, and its ability to adapt to diverse environmental situations, this bacterium affords an excellent model system to study global metabolic processes. It appears that *P. fluorescens* reprograms its metabolism in an effort to utilize citrate in an anaerobic fashion. Faced with an ineffective ACN, the organism up-regulates CL, a stratagem designed to bypass the TCA cycle and oxidative phosphorylation. The subsequent generation of the high-energy metabolite phosphoenolpyruvate (PEP) allows for ATP synthesis via substrate-level phosphorylation. The pivotal role of metabolism in the adaptation to nitrosative stress and its significance in countering RNS-resistant bacteria are discussed.

## Materials and Methods

### Bacterial culture and isolation of cellular fractions

The bacterial strain *P. fluorescens,* ATCC 13525, was maintained (on 2% agar) and grown in a phosphate mineral medium containing Na_2_HPO_4_ (6 g), KH­_2_PO_4_ (3 g), MgSO_4_.7H_2_O (0.2 g), NH_4_Cl (0.8 g), and citric acid (4 g) per litre of distilled and deionized H_2_O. Trace elements were added. Nitrosative stress was induced via the addition of 1, 5, 10, 15 or 20 mM of sodium nitroprusside (SNP), respectively [5]. Control cultures had no added SNP and/or contained 10 mM sodium ferrocyanide (SFC). The latter has a similar composition to SNP except for the absence of the nitroso functional group and hence is unable to act as a source of NO. For select experiments, nitrosative stress was initiated by the addition of 1 mM diethylamine NONOate (DEANO), in an effort to ascertain if indeed NO was the stressor and not any other component of SNP. The only similarity between SNP and DEANO is their ability to generate NO. The pH was adjusted to 6.8 with dilute NaOH. The media was then dispensed in aliquots of 200 mL into 500 mL Erlenmeyer flasks with foam plugs and autoclaved for 20 min at 121°C. Innoculations were made with 1 mL of stationary phase cells grown in a control culture and aerated on a gyratory water bath shaker (Model 76; New Brunswick Scientific). To ensure an ongoing nitrosative response, a second dose of DEANO was introduced after 16 hours of growth. Cells and spent fluids were isolated at various growth phases.

*P. fluorescens* cells were pelleted by centrifugation at 10,000 x g for 10 min at 4°C. After a washing with 0.85% NaCl, cells were resuspended in cell storage buffer consisting of 50 mM Tris-HCl, 5 mM MgCl_2_ and 1 mM phenylmethylsulphonyl fluoride (pH 7.3). Cells were disrupted ultrasonically and then centrifuged at 3,000 x g for 30 min at 4°C to remove intact bacteria. The supernatant was collected and centrifuged at 180,000 x g for 3 h, resulting in a soluble cell free extract (CFE) and a membrane CFE. The soluble fraction was further centrifuged at 180,000 x g for 1 to obtain a membrane-free system. The Bradford assay [8] was performed in triplicate in order to quantify the protein contents and bovine serum albumin was used as the standard. Purity of the fractions was ascertained as described in [9]. These CFE fractions were kept at 4°C for up to 5 days or frozen at −20°C for storage up to a maximum of 4 weeks. The pH of the spent fluid was also recorded.

### Metabolomic studies in cell-free extracts: selective inhibition of PEPC

Spent fluid and soluble CFE from control and SNP-stressed cells were used to determine various metabolite levels via high performance liquid chromatography (HPLC). Two mg/mL of protein equivalent of soluble CFE was heated gently for 10 minutes to precipitate proteins and lipids and analyzed by HPLC as described in [10]. To verify the disparate citrate metabolism, 2 mg/mL protein equivalent of soluble CFE was incubated for 2 hrs at 26°C in a reaction buffer (25 mM Tris, 5 mM MgCl_2_; pH 7.0) containing 2 mM citrate and 0.5 mM nicotinamide adenine dinucleotide phosphate (NADP). To confirm the importance of PEPC in the metabolic adaptation to SNP, 2 mg/mL protein equivalent of soluble CFE was incubated for 30 min in reaction buffer containing 2 mM citrate, 1.0 mM P_i_ and 0.5 mM AMP with or without a 1∶500 dilution of antibody against PEPC. Chemical shifts were referenced to standard compounds and internal standards under the same conditions and the peaks were quantified using the Empower Software (Waters Corporation). The HPLC was standardized using a five-point calibration protocol prior to each injection. The relative quantities of nitrite and nitrate in the various cellular components were monitored by Griess reaction [11].

### Blue Native PAGE and in-gel enzyme activity staining

Blue Native (BN) polyacrylamide gel electropohoresis (PAGE) and in-gel activity staining were performed as described previously [10], [12]. Soluble proteins from *P. fluorescens* were prepared in a native buffer (50 mM Bis-Tris, 500 mM ε-aminocaproic acid, pH 7.0, 4°C) at a final concentration of 4 mg of protein per ml. Membrane proteins were prepared in a similar manner except 1% (v/v) β-dodecyl-D-maltoside was added to the preparation to facilitate the solubilization of the membrane-bound proteins. To ensure optimal protein separation, 4–16% linear gradient gels were cast with the Bio-Rad MiniProtean™ 2 system using 1 mm spacers. Sixty micrograms of protein from soluble or membrane CFE was loaded into the wells. Gels were electrophoresed as described in [13]. Following electrophoresis, gel slabs were equilibrated for 15 min in a reaction buffer (25 mM Tris-HCl, 5 mM MgCl2, at pH 7.4). Enzyme activities were detected in the gel by the reduction and precipitation of iodonitrotetrazolium (INT) as formazan.

ACN activity was visualized using a reaction mixture consisting of a reaction buffer, 5 mM citrate, 0.5 mM NADP, 5 units/mL isocitrate dehydrogenase (Sigma), 0.2 mg/mL phenazine methosulfate (PMS) and 0.4 mg/mL of INT. Tricarballylic acid (1 mM) was utilized to maintain the stability of ACN [14]. NADP-dependent isocitrate dehydrogenase (ICDH-NADP) activity was visualized using a reaction mixture containing 5 mM isocitrate, 0.5 mM NADP, 0.2 mg/mL PMS and 0.4 mg/mL INT. The same reaction mixture was utilized for ICDH-NAD except 0.5 mM nicotinamide adenine dinucleotide (NAD) was utilized in lieu of NADP. The activity band for isocitrate lyase (ICL) was made apparent using reaction buffer, 5 mM isocitrate, 0.5 mM NAD, 10 units of lactate dehydrogenase (LDH), 0.2 mg/mL of PMS and 0.4 mg/mL of INT. Complex 1 was detected by the addition of 5 mM NADH, and 0.4 mg/mL INT. Succinate dehydrogenase (SDH) was monitored by the addition of 60 mM succinate, 5 mM KCN, 0.2 mg/mL of PMS and 0.4 mg/mL of INT [15]. Complex IV was assayed by the addition of 10 mg/mL of diaminobenzidine, 10 mg/mL cytochrome C, and 562.5 mg/mL of sucrose. 5 mM KCN was added to the reaction mixture to confirm the identity of Complex IV [16]. Malate dehydrogenase (MDH) was assayed by the addition of 5 mM malate, 0.5 mM NAD, 0.2 mg/mL of PMS and 0.4 mg/mL of INT. The activity of acetate kinase was assessed with a reaction mixture of 5 mM acetyl phosphate, 1 mM ADP, 5 mM glucose, 10 units of glucose-6-phosphate dehydrogenase (G6PDH), 10 units hexokinase, 1 mM NADP, 0.2 mg/mL of PMS and 0.4 mg/mL of INT.

Pyruvate dehydrogenase (PDH) was detected utilizing a reaction mixture consisting of 5 mM pyruvate, 0.1 mM CoA, 0.5 mM NAD, 0.2 mg/mL of PMS and 0.4 mg/mL of INT. LDH was assayed using a reaction mixture containing 5 mM lactate, 0.5 mM NAD, 0.2 mg/mL of PMS and 0.4 mg/mL of INT. For NAD(P)-producing enzymes PMS was replaced by 2,4-dichloroindophenol (DCIP) as the electron coupler [13]. The activities of CL, phosphoenolpyruvate carboxykinase (PEPCK) and pyruvate carboxylase (PC) were ascertained by utilizing enzyme-coupled assays as described in [13]. PEPC was assayed in a similar manner as PEPCK except no ADP was present in the mixture. Pyruvate kinase (PK) was probed as described in [17]. PPDK was monitored using a reaction mixture consisting of 5 mM PEP, 0.5 mM AMP, 0.5 mM sodium pyrophosphate, 0.5 mM NADH, 10 units of LDH, 0.0167 mg/mL of DCIP and 0.4 mg/mL of INT. S-nitrosoglutathione (GSNO) was freshly prepared by adding 1 mL of a 100 mM solution of reduced glutathione (GSH) to 1mL of a 100 mM solution of sodium nitrite (in 0.1% HCl) [18]. The concentration of GSNO was determined (ε = 908 M^−1^ cm^−1^ at 334 nm) and a reaction mixture consisting of 5 mM GSNO, 0.5 mM NADPH, 0.0167 mg/mL of DCIP and 0.4 mg/mL of INT was prepared in order to determine the in-gel activity. All the enzymes were probed individually in separate gels.

The activities of NR and NIR was verified using a reaction mixture consisting of 5 mM sodium nitrate or 5 mM sodium nitrite, respectively, 0.5 mM NADPH, 0.0167 mg/mL of DCIP and 0.4 mg/mL of INT. Once bands reached their desired intensities, reactions were halted using destaining solution (40% methanol, 10% glacial acetic acid). Band specificity was ascertained by performing activity stains in the absence of substrate. Activity bands were quantified using SCION Image for Windows (SCION Imaging Corp.). Proper protein loading was determined by Coomassie staining for total proteins. Select activity bands were routinely cut and incubated with their respective substrates. The metabolite profiles were monitored by HPLC. Unless otherwise mentioned, all comparative experiments were performed at the late logarithmic phase of growth.

### 2D SDS-PAGE and protein expression

For 2D SDS-PAGE, the activity bands for CL were excised and soaked for 30 min in a solution of 1% (w/v) SDS and 1% (v/v) 2-mercaptoethanol. The bands were rinsed twice for 10 s with SDS-PAGE electrophoresis buffer (25 mM Tris-HCl, 192 mM glycine and 0.1% (w/v) SDS; pH 8.3) then placed vertically in the wells of a 10% SDS gel (8×7 cm). Electrophoresis was conducted at 80 V at room temperature until the proteins reached the separating gel. The voltage was then raised to 200 V until completion. Upon completion of the SDS-PAGE, gels were stained with the Bio-Rad Silver Staining Kit.

### Recovery experiments

To ascertain that nitrosative stress was indeed triggering these metabolic changes, the SNP-treated cells grown to late logarithmic phase were transferred into control medium (without SNP). Similar experiments were performed with control cells transferred to SNP-containing medium. Following an 8 h incubation period, the cells were harvested, and CFE was assayed for protein concentration and enzymatic activity.

### In-cell Western blot analyses

In-cell Western assays were modified from the Odyssey® Infrared Imaging System protocol document (Li-cor doc# 988-08599). Briefly, *P.fluorescens* was seeded in 96-well plates from a pre-culture for 8 h and treated with control, 5 mM SNP, or 10 mM SNP conditions for 24 h. Recovery experiments were performed as described previously. After treatment, the media was removed and the cells were washed thrice with phosphate buffered saline (PBS) [136.8 mM NaCl, 2.5 mM KCl, 1.83 mM Na_2_HPO_4_, and 0.4313 mM KH_2_PO_4_ at pH 7.4]. The cells were then fixed with 37% formaldehyde for 20 min at room temperature. The fixing solution was then removed and the cells were rinsed with PBS containing 0.1% tween-20. Blocking ensued using the Odyssey® blocking buffer for 2 . Primary antibody incubations occurred over a 1 h period with gentle shaking. Mouse monoclonal anti-NO-tyrosine (Abcam), rabbit polyclonal anti-PEPC (Abcam) and rabbit polyclonal anti-glutamate dehydrogenase (GDH) were all diluted to a concentration of 1∶200 in blocking buffer. GDH was utilized as an internal standard for this assay as its expression did not vary significantly with the culture conditions. Secondary antibodies consisted of donkey anti-mouse IR 680 (Li-cor; red) and goat anti-rabbit IR-780 (Li-cor; green) diluted to 1∶1000. Relative levels were compared to an in-cell Bradford assay. The infrared signal as well as the signal from the Bradford reagent was detected using an Odyssey® Infrared Imager (Li-cor).

#### Statistical analysis

Data were expressed as means ± standard deviations. Statistical correlations of the data were checked for significance using the Student t test (p≤0.05). All experiments were performed at least twice and in triplicate.

## Results

### Bacterial growth, RNS stress and citrate consumption

**Figure 1:**
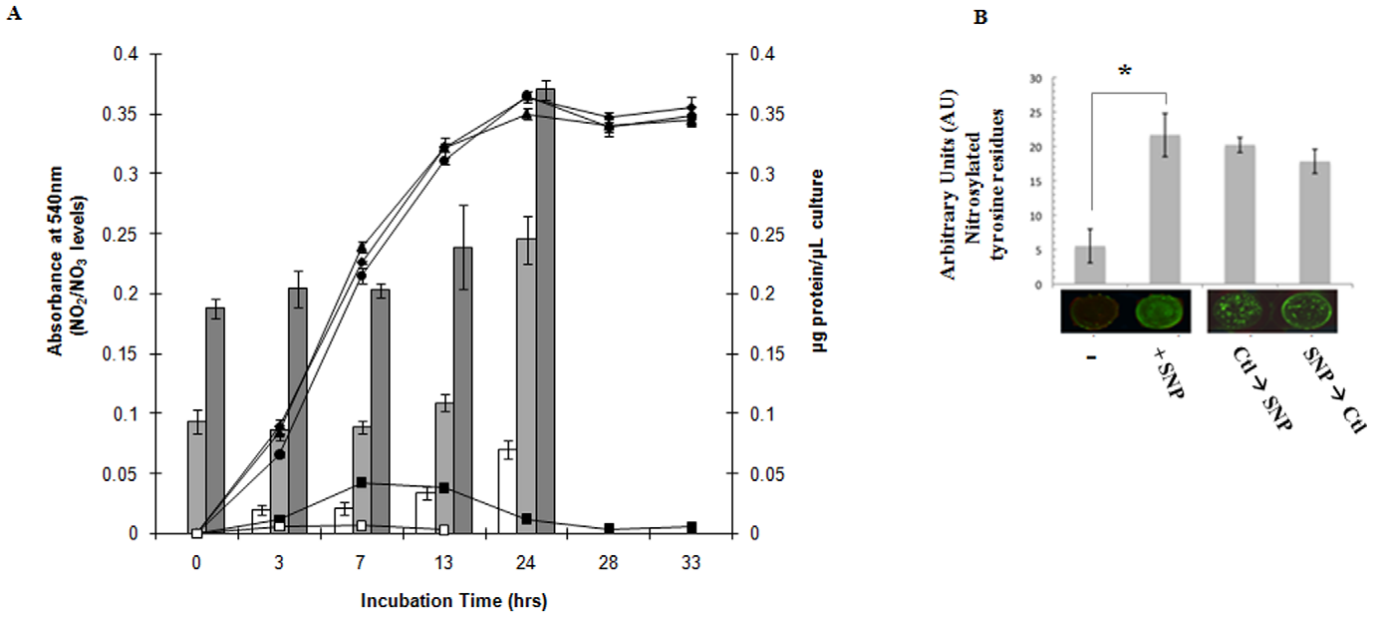
Nitrosative stress in *P. fluorescens*. *A,* cellular yield: ▴, control cultures; ♦, 5 mM SNP-containing cultures; •, 10 mM SNP-containing cultures; ▪, 15 mM SNP-containing cultures; □, 20 mM SNP-containing cultures. Nitrite/nitrate levels in spent fluids as measured by their absorbance at 540 nm after the Griess assay: □, control cultures; ▪, 5 mM SNP-containing cultures; ▪, 10 mM SNP-containing cultures. *B*, In cell Western blot analysis of *P. fluorescens* under nitrosative stress. *P. fluorescens* was grown to late logarithmic phase in 96 well plates and then treated with control (−) and 10 mM SNP conditions for 24 h (+SNP). Following treatment, control cells were placed in media containing 10 mM SNP for 8 h (Ctl→SNP) and 10 mM SNP-treated cells were recovered with control media for 8 h (SNP→Ctl). Total protein concentration was assessed by the Bradford assay. The cells were then analyzed for expression of nitrosylated tyrosine residues by quantification of infrared fluorescence (n = 3. *p≤0.01, mean ± standard deviation).

*P. fluorescens* survived in a 10 mM SNP-stressed environment with minimal change in growth rate or cellular yield in comparison to cells grown in the control media (Fig. 1). Cultures subjected to concentrations of 15 and 20 mM SNP showed severely retarded growth and no growth at all, respectively. It may be within the realm of possibilities that at these higher concentrations the physico-chemical properties of SNP in the medium utilized were conducive to the easier release of the toxicant. As SNP is known to liberate NO [19], it was critical to verify that this gaseous radical was being released and detoxified to the relatively innocuous nitrite/nitrate (NO_2_/NO_3_). An increase in the concentration of NO_2_ and NO_3_ in 5 and 10 mM SNP-stressed cells relative to the control was observed (Fig. 1A). In the soluble CFE isolated from the 10 mM SNP cultures, there was 6 fold more NO_2_/NO_3_ than the control (data not shown). Furthermore, while in the presence of SFC, no significant change in NO_2_ or NO_3_ was evident, in the DEANO-treated cells, these moieties were relatively higher (data not shown). Nitrosylated proteins were also more abundant in the stressed cell. Indeed, Western blot with nitrotyrosine antibody helped detect higher levels of this residue in the cells subjected to nitrosative stress (Fig. 1B). Thus, it was evident the toxicity of SNP was being felt by the microbe.

### The RNS detoxifying enzymes

**Figure 2:**
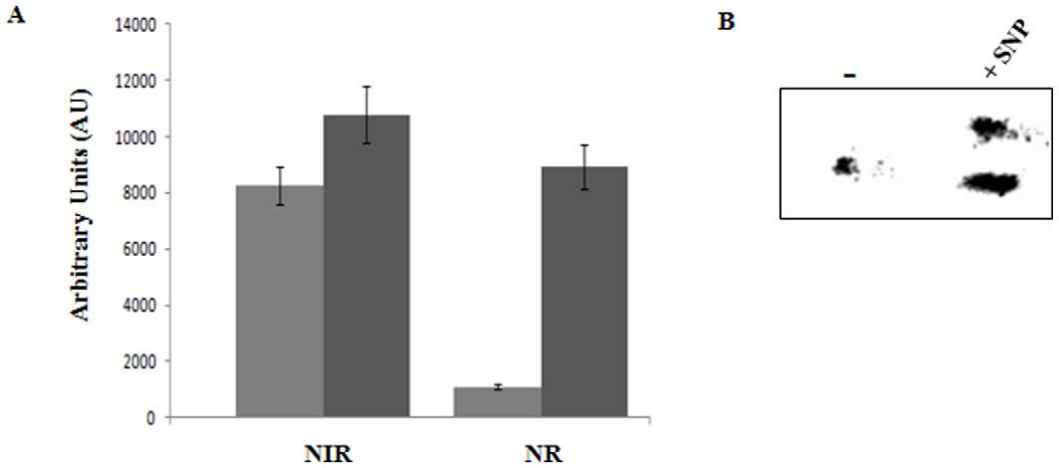
RNS detoxifying mechanisms in *P. fluorescens*. *A*, BN-PAGE analysis of NIR and NR from membrane CFE: ▪, control cultures; ▪, 10 mM SNP-treated cultures. Bands were quantified using SCION Imaging Software (n = 3, mean ± standard deviation). *B*, activity staining of GSNOR in soluble CFE (−, control cultures; *+*SNP, 10 mM SNP-treated cultures).

The protection against RNS has been shown to be mediated by a variety of enzyme systems involved in the direct conversion of NO into innocuous products and the denitrosylation of nitrosylated proteins [5]. To ascertain if these detoxification strategies were invoked by *P. fluorescens* subjected to SNP, we probed NIR and NR. The activities of these enzymes were monitored by BN-PAGE and were found to be elevated in the SNP-stressed cells compared to the control cells (Fig. 2A). GSNOR, an enzyme that generates reduced glutathione was identified as two isoforms in the *P. fluorescens* challenged by nitrosative stress. Their activities were markedly higher compared to the control cells (Fig. 2B). Catalase, a detoxifying enzyme involved in the regulation of peroxide levels, was found to be 2 fold higher in activity in the SNP-treated cells (data not shown).

### The tricarboxylic acid cycle and oxidative phosphorylation

**Figure 3:**
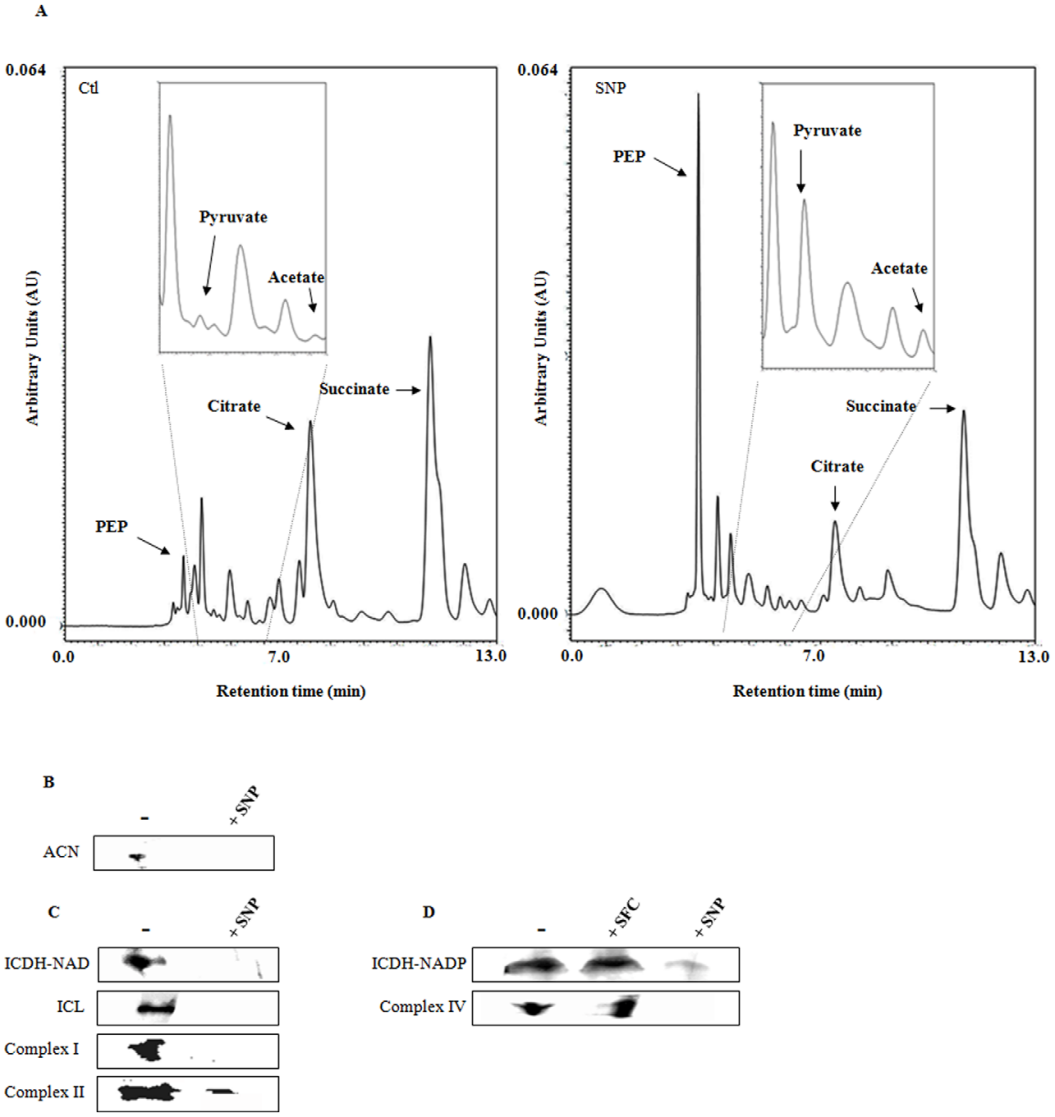
Influence of NO on the TCA cycle. *A,* Representative chromatographs showing metabolite levels in soluble CFE from *P. fluorescens* grown in control and 10 mM SNP-containing cultures. *B,* In-gel activity analysis for ACN. *C*, in-gel activity staining of key metabolic enzymes. *D*, activity stains with sodium ferrocyanide as a control (−, control cultures; *+*SNP, 10 mM SNP-treated cultures; +SFC, 10 mM SFC-treated cultures).

As citrate was the sole source of carbon in this defined medium, it was important to verify how this tricarboxylic acid was metabolized in the control and SNP-stressed cells. The cells subjected to nitrosative stress had significantly more PEP, pyruvate and acetate than the control cells while levels of citrate, succinate and α-ketoglutarate were found to be lower in the stressed CFE (Fig. 3A). The identities of these metabolites were further confirmed by spiking the samples with known standards. The heterogeneity of these metabolic profiles prompted us to examine the enzymatic adjustments that would provoke such a change.

Since nitrosative stress is known to affect Fe-S clusters [20], it was important to evaluate ACN, a critical gate-keeper of the tricarboxylic acid cycle. It isomerizes citrate into isocitrate with the aid of a 4Fe-4S cluster located in its active site [20]. The stressed cells did not show any activity band (Fig. 3B) As reported before, a sharp band was evident in the control [14], [15], [21]. This finding led us to probe enzymes further downstream in the TCA cycle that might be able to compensate for this shortcoming.

Two isocitrate metabolizing enzymes, ICDH-NAD and ICL, were shown to be markedly decreased as indicated by the lack of a formazan band in the lane containing proteins from the stressed fraction (Fig. 3C). The activity of ICDH-NADP, a cytoplasmic enzyme which produces NADPH and α-ketoglutarate, was also found to be significantly downregulated in the SNP-stressed culture compared to the control culture (Fig. 3D). Indeed, in the presence of NO, key cysteine and tyrosine residues in this enzyme have been shown to be modified [22]. The activity of ICDH-NADP was preserved in *P. fluorescens* treated with 10 mM SFC, a chemical analog to SNP not containing the nitroso functional group (Fig. 3D). As the TCA cycle was perturbed, it was important to evaluate the status of oxidative phosphorylation, a Fe-S cluster rich enzyme network dedicated to the production of ATP. All the complexes (I, II, IV) that were monitored were found to be ineffective. Activity bands indicative of these proteins were barely discernable in the membrane CFE from the SNP-stressed cells (Fig. 3C) [the activity of complex IV was preserved in *P. fluorescens* treated with 10 mM sodium ferrocyanide (Fig. 3D)]. These bands reappeared when the RNS-stressed cells were incubated in the control media. These enzymes were similarly affected in the DEANO treated cells (data not shown).

### CL activity, expression and regulation

**Figure 4:**
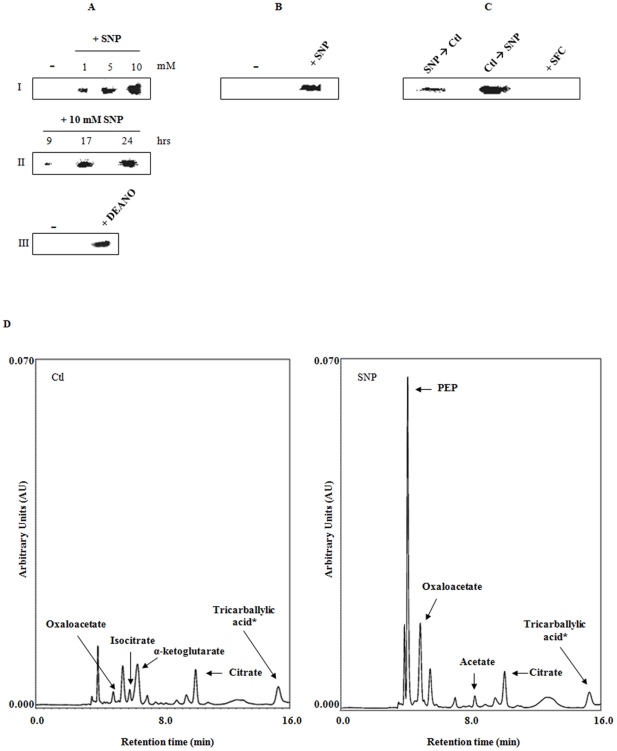
Activity and expression analysis of CL from *P. fluorescens*. *A,* in-gel CL activity in soluble CFE isolated from *P. fluorescens*. **I**) The dose effect of SNP on CL activity **II**) Time dependence of CL activity in cultures treated with 10 mM SNP. **III**) CL activity in media containing 1 mM DEANO (−, control cultures; +DEANO, 1 mM DEANO-treated cultures). *B*, 2D SDS-PAGE (−, control cultures; *+*SNP, 10 mM SNP-treated cultures). *C*, regulation of CL activity by SNP. 10 mM SNP-treated cells were grown to late logarithmic phase then transferred to control media for 8 h and vice versa. *D*, HPLC analysis of CL activity. Soluble CFE from cells grown in control and 10 mM SNP-treated media were given 5 mM citrate and 0.5 mM NADP for 2 hrs (*: tricarballylic acid (1 µM) as internal standard).

The apparent inability of SNP-stressed cultures to metabolize citrate via the TCA cycle prompted us to evaluate other alternative pathways for citrate consumption. The presence of elevated levels of acetate in the SNP-stressed cells pointed toward a possible role of CL. This enzyme can cleave citrate into oxaloacetate and acetate without the participation of ATP and coenzyme A [23], [24]. In this instance, CL generated oxaloacetate and acetate in the absence of ATP and coenzyme A. It increased in a time-dependent manner in the SNP-stressed cultures (Fig. 4A). CL was also found to be modulated by the levels of SNP in the medium (Fig. 4A). To confirm that the increased activity of this enzyme was the result of NO and not other components of SNP, the activity was monitored in cells treated with DEANO and found to be up-regulated in these cultures (Fig. 4A). Furthermore, CL was not visible in cells subjected to 5 mM NO_2_ or NO_3_, indicating that this is a NO-mediated occurrence (data not shown). BN-PAGE followed by 2D SDS-PAGE revealed that the expression of this enzyme was up-regulated in the SNP-stressed cells (Fig. 4B). In an effort to prove that nitrosative stress was indeed promoting the up-regulation of this enzyme, control cells were transferred in the stressed medium and stressed cells in the control cultures. A reverse trend in the activity of CL was observed (Fig. 4C). When the soluble CFE from both control and stressed were incubated with citrate respectively, oxaloacetate and acetate was observed only in the latter. However, in the presence of NADP, the control CFE readily produced α-ketoglutarate and NADPH (Fig. 4D). These observations clearly demonstrated that CL was an important component of the metabolic reconfiguration evoked by nitrosative stress in *P. fluorescens*.

### Oxaloacetate and pyruvate metabolism: The role of PEPC

**Figure 5:**
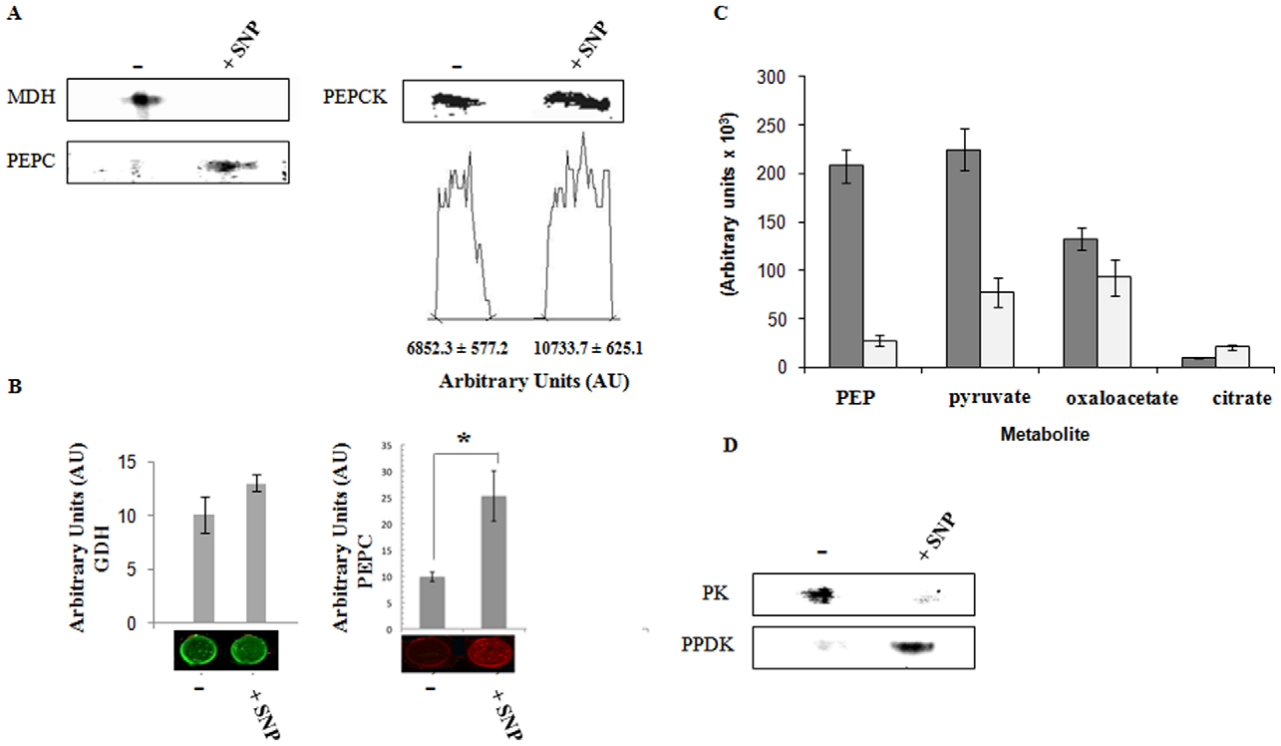
Oxaloacetate and PEP metabolism. *A*, BN-PAGE analysis of oxaloacetate metabolizing enzymes. Bands were quantified using SCION Imaging Software (n = 3± standard deviation). *B*, In-cell Western blot analysis. *P.fluorescens* was grown to late logarithmic phase in 96 well plates then treated with control (−) and 10 mM SNP conditions for 24 h (+SNP). The cells were then analyzed for **(**GDH) and (PEPC) and expression was quantified by infrared fluorescence (n = 5, mean ± standard deviation. *p≤0.01). C, Citrate consumption in *P. fluorescens* exposed to ntirosative stress. Soluble CFE from 10 mM SNP-treated cells was incubated with 2 mM citrate, 1.0 mM Pi and 0.5 mM AMP for 30 min without (▪) and with an antibody (1∶500 dilution) against PEPC (□). n = 3, mean ± standard deviation. D, in-gel activity stain of the pyruvate generating enzymes PK and PPDK (−, control cultures; *+*SNP, 10 mM SNP-treated cultures).

As oxaloacetate was readily available in the SNP-stressed cells due to the upregulation of CL, it was important to decipher how this dicarboxylic acid was being metabolized. PEPC, PEPCK and MDH can all interact with oxaloacetate. The activity of MDH was found to be lower in the SNP-stressed system (Fig. 5A). However, unexpectedly, the PEPC activity that was elevated in the *P. fluorescens* challenged with SNP was barely discernable in the untreated cultures (Fig. 5A). This enzyme mediates the conversion of oxaloacetate in the presence of inorganic phosphate into the high energy PEP [25]. Western blots performed in these cells demonstrated that the increase in PEPC activity in stressed bacteria was directly correlated to increased expression of this protein (Fig. 5B). PEPCK, the other enzyme that transforms oxaloacetate into PEP with the participation of ATP or GTP was also elevated in the cells subjected to nitrosative stress (Fig. 5A). Hence these two enzymes were aiding the production of the high energy PEP, a metabolite that was abundant in the stressed cells. The production of PEP was found to be drastically reduced in SNP-stressed CFE treated with an antibody against PEPC. Although citrate was consumed, albeit to a lesser extent compared to the reaction without antibodies, the formation of PEP, a key metabolite essential in the production of ATP and pyruvate was severely affected (Fig. 5C).

**Figure 6:**
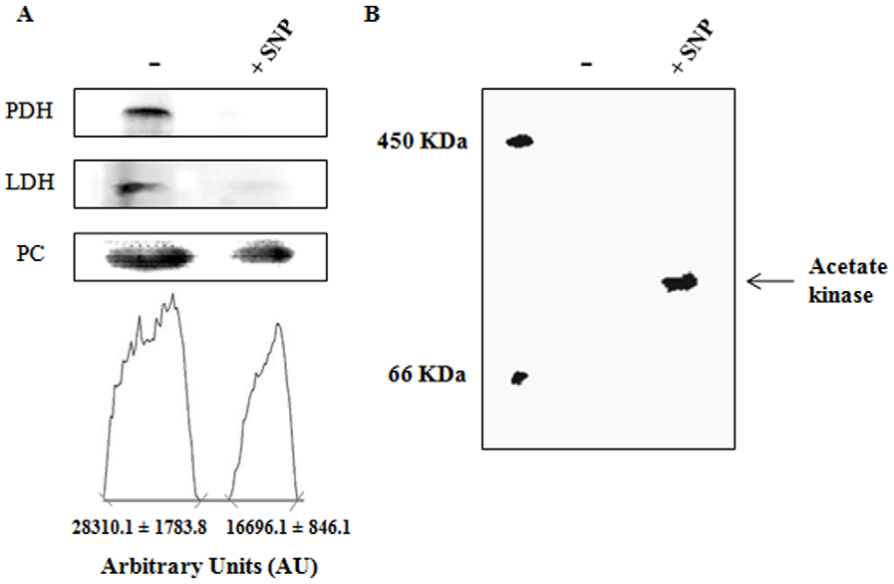
Pyruvate and acetate utilizing enzymes. *A,* BN-PAGE analysis of pyruvate metabolizing enzymes. Bands were quantified using SCION Imaging Software (n = 3± standard deviation). *B*, activity staining of acetate kinase in membrane CFE (−, control cultures; *+*SNP, 10 mM SNP-treated cultures). BSA and ferritin were utilized as molecular mass markers to ensure proper migration.

The presence of increased pyruvate and acetate in the soluble CFE from the stressed cells coupled with the enhanced production of PEP directed our attention towards the homeostasis of pyruvate. PK and PPDK are the two main participants in the conversion of PEP into pyruvate with the concomitant formation of ATP. The former utilizes ADP while the latter invokes the participation of AMP and PP_i_ as the cofactors [26]. Interestingly, the activity of PK was lower in the RNS-challenged environment, while the activity of PPDK was sharply increased compared to the control cells (Fig. 5D). This enzymatic manipulation would indeed allow RNS-challenged *P. fluorescens* to generate ATP more efficiently. Indeed, when soluble CFE from SNP or DEANO-treated cells was incubated with oxaloacetate and AMP, ATP and pyruvate were readily formed (data not shown). As pyruvate and acetate were seemingly the end products of this metabolic reconfiguration, it was important to analyze enzymes that may be involved in their consumption. The activities of PDH, LDH and PC were all found to be lower in *P. fluorescens* exposed to nitrosative stress (Fig. 6A). This would indeed result in the pooling of pyruvate as seen in the soluble CFE. The activity of acetate kinase was found to be up-regulated in the treated cells (Fig. 6B), indicating a role for acetate in energy storage.

## Discussion

Although the involvement of enzymes mediating the detoxification of RNS has been widely reported, this study is the first demonstration of the pivotal role metabolism plays in combating nitrosative stress. The microbe utilizes its various metabolic networks to not only circumvent the toxic impact of RNS but also to generate energy via substrate-level phosphorylation. There is a body of literature detailing the different heme enzymes that participate in the formation of relatively non-toxic nitrate. In *E. coli*, both flavohemoglobin and a cytochrome C nitrate reductase have been implicated in the elimination of NO as NO_3_ [5]. In this instance, it appears that *P. fluorescens* does indeed utilize both nitrite and nitrate reductase to perform this task. Catalase, an enzyme involved in the detoxification of H_2_O_2_ was upregulated in the cells exposed to nitrosative stress. This is in accordance with numerous studies demonstrating the elevation of catalase activity in organisms subjected to RNS [27], [28]. GSNOR is an important enzyme that contributes to the survival of organisms subjected to nitrosative stress associated with the innate immune response of the human body [4]. The two isoforms that showed increased activities in the SNP-challenged *P. fluorescens* may be indeed aimed at minimizing S-nitrosothiols and S-nitrosylated proteins, hallmarks of nitrosative stress.

The participation of these classical RNS-detoxifying enzymes in fending off nitrosative stress was not surprising. However, the metabolic reprogramming aimed at circumventing RNS that was observed in *P. fluorescens* is indeed a seminal finding, a phenomenon that has yet to be fully delineated. Faced with citrate, the sole source of carbon and a RNS-induced ineffective ACN and ICDH, the microbe has to find an alternative pathway to degrade citrate if it is to survive. In this instance, an ATP and coenzyme A independent CL was invoked. This enzyme mediates the formation of oxaloacetate and acetate and is common in microbial systems subjected to anaerobiosis [29]. This is not surprising as RNS severely damage the ETC and limit aerobic respiration [30]. In our study, the three ETC complexes that were probed were found to be markedly ineffective. Furthermore, the inactivation of ICDH and αKGDH would impede the functioning of the TCA cycle, an adaptation critical for survival under nitrosative stress. Although ACN was found to be ineffective, it may be quite possible that this enzyme that exists in three isoforms was not detected under our experimental conditions [31].

The dicarboxylic acid, oxaloacetate was subsequently metabolized to PEP by PEPC. This enzyme primarily has an anaplerotic role in the production of oxaloacetate. However, in this study as the microbe was supplied with citrate, the sole carbon source, oxaloacetate was readily available due to upregulation of CL. Hence, it is quite conceivable that this dicarboxylic acid may be an important source of PEP. Although PEPCK with enhanced activity was evident in the RNS-stressed cells, PEPC was more prominent. The latter enzyme was not discerned in the control cultures. The inability of PEPC-inhibited CFE to generate PEP effectively suggests that this enzyme is critical in this adaptation process. The production of PEP from oxaloacetate may also have been favored due to the close proximity of this protein with PPDK, an enzyme mediating the synthesis of pyruvate and ATP with AMP and PPi as cofactors. These enzymes migrate in association with CL, as observed by BN-PAGE. The incubation of this band did generate all the products with citrate, AMP and pyrophosphate (PP_i_) as substrates (data not shown).

The presence of elevated amounts of acetate in the soluble CFE from the stressed cells directed our attention to a possible role of pyruvate in the non-enzymatic neutralization of RNS. Pyruvate has been shown *in vivo* to react with RNS to liberate acetate [32]. Furthermore, there is a body of literature on the utilization of pyruvate and other ketoacids as anti-oxidants [33], [34]. We have recently demonstrated the role of αKG in the scavenging of ROS with the concomitant formation of succinate [10], [35]. The elevated levels of acetate kinase and the accumulation of pyruvate from citrate metabolism would suggest that *P. fluorescens* may be utilizing this keto acid to quell oxidative and nitrosative stress. However, these postulations have to await further investigation.

**Figure 7:**
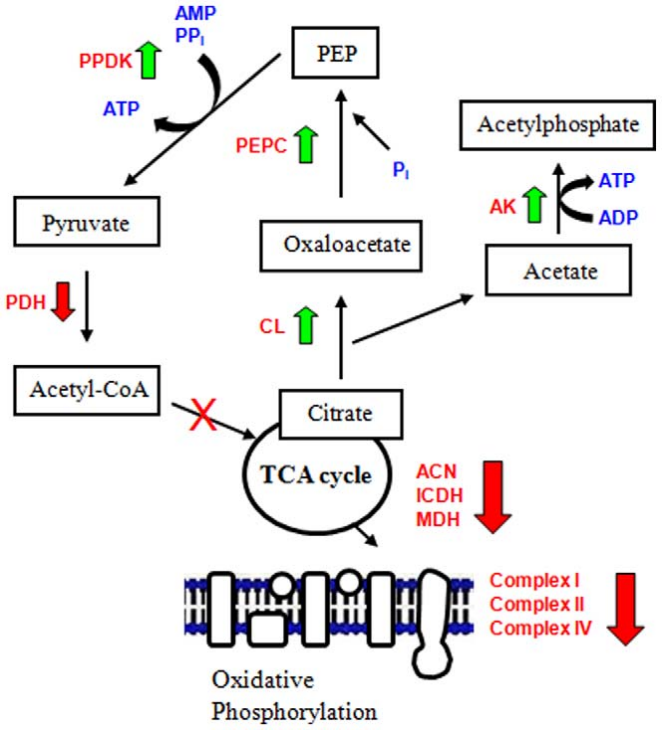
Citrate metabolism in *Pseudomonas fluorescens* under nitrosative stress. Increased activity and expression of CL allows for the degradation of citrate and circumvention of nitrosative stress via a novel metabolic network in *P. fluorescens* subjected to SNP (⇓, decreased activity, ⇑, increased activity). CL, citrate lyase; AK, acetate kinase; ICDH, isocitrate dehydrogenase; MDH, malate dehydrogenase; PEPC, phosphoenolpyruvate carboxylase; PPDK, pyruvate phosphate dikinase; PDH, pyruvate dehydrogenase.

In conclusion, our findings reveal an intriguing role of metabolism in the adaptation of *P. fluorescens* to a nitrosative environment (Fig. 7). Although the classical RNS-detoxifying enzymes were invoked, the microbe also completely reprogrammed its metabolic networks to survive this toxic challenge. The noxious impact of RNS on the TCA cycle and oxidative phosphorylation was mitigated by the upregulation of CL, an enzyme that degraded citrate into oxaloacetate and acetate. The dicarboxylic acid subsequently provided the two ingredients, pyruvate and ATP, that ensured the survival of *P. fluorescens* under the insult of SNP. Thus, metabolic changes are the underlying force at the centre of most, if not all, molecular adaptation phenomena and necessary in the anti-RNS defense strategy in this microbe. Targeting the critical components of these metabolic networks may provide a potent tool against RNS-resistant bacteria.

## References

[1] Pun PB, Lu J, Kan EM, Moochhala S (2010). Gases in the mitochondria.. Mitochondrion.

[2] Durzan DJ, Pedroso MC (2002). Nitric oxide and reactive nitrogen oxide species in plants.. Biotechnol Genet Eng Rev.

[3] Emre Y, Nubel T (2010). Uncoupling protein UCP2: when mitochondrial activity meets immunity.. FEBS Lett.

[4] Benhar M, Forrester MT, Stamler JS (2009). Protein denitrosylation: enzymatic mechanisms and cellular functions.. Nat Rev Mol Cell Biol.

[5] Poole RK (2005). Nitric oxide and nitrosative stress tolerance in bacteria.. Biochem Soc Trans.

[6] van Wonderen JH, Burlat B, Richardson DJ, Cheesman MR, Butt JN (2008). The nitric oxide reductase activity of cytochrome c nitrite reductase from *Escherichia coli*.. J Biol Chem.

[7] McLean S, Bowman LA, Sanguinetti G, Read RC, Poole RK (2010). Peroxynitrite toxicity in *Escherichia coli* K12 elicits expression of oxidative stress responses and protein nitration and nitrosylation.. J Biol Chem.

[8] Bradford MM (1976). A rapid and sensitive method for the quantitation of microgram quantities of protein utilizing the principle of protein-dye binding.. Anal Biochem.

[9] Singh R, Lemire J, Mailloux RJ, Appanna VD (2008). A novel strategy involved in [corrected] anti-oxidative defense: the conversion of NADH into NADPH by a metabolic network.. PLoS One.

[10] Mailloux RJ, Singh R, Brewer G, Auger C, Lemire J (2009). Alpha-ketoglutarate dehydrogenase and glutamate dehydrogenase work in tandem to modulate the antioxidant alpha-ketoglutarate during oxidative stress in *Pseudomonas fluorescens*.. J Bacteriol.

[11] Miranda KM, Espey MG, Wink DA (2001). A rapid, simple spectrophotometric method for simultaneous detection of nitrate and nitrite.. Nitric Oxide.

[12] Schagger H, von Jagow G (1991). Blue native electrophoresis for isolation of membrane protein complexes in enzymatically active form.. Anal Biochem.

[13] Singh R, Chenier D, Beriault R, Mailloux R, Hamel RD (2005). Blue native polyacrylamide gel electrophoresis and the monitoring of malate- and oxaloacetate-producing enzymes.. J Biochem Biophys Methods.

[14] Middaugh J, Hamel R, Jean-Baptiste G, Beriault R, Chenier D (2005). Aluminum triggers decreased aconitase activity via Fe-S cluster disruption and the overexpression of isocitrate dehydrogenase and isocitrate lyase: a metabolic network mediating cellular survival.. J Biol Chem.

[15] Mailloux RJ, Hamel R, Appanna VD (2006). Aluminum toxicity elicits a dysfunctional TCA cycle and succinate accumulation in hepatocytes.. J Biochem Mol Toxicol.

[16] Lemire J, Mailloux R, Appanna VD (2008). Zinc toxicity alters mitochondrial metabolism and leads to decreased ATP production in hepatocytes.. J Appl Toxicol.

[17] Mailloux RJ, Appanna VD (2007). Aluminum toxicity triggers the nuclear translocation of HIF-1alpha and promotes anaerobiosis in hepatocytes.. Toxicol In Vitro.

[18] Gibson A, Babbedge R, Brave SR, Hart SL, Hobbs AJ (1992). An investigation of some S-nitrosothiols, and of hydroxy-arginine, on the mouse anococcygeus.. Br J Pharmacol.

[19] Pelletier AM, Venkataramana S, Miller KG, Bennett BM, Nair DG (2010). Neuronal nitric oxide inhibits intestinal smooth muscle growth.. Am J Physiol Gastrointest Liver Physiol.

[20] Tortora V, Quijano C, Freeman B, Radi R, Castro L (2007). Mitochondrial aconitase reaction with nitric oxide, S-nitrosoglutathione, and peroxynitrite: mechanisms and relative contributions to aconitase inactivation.. Free Radic Biol Med.

[21] Chenier D, Beriault R, Mailloux R, Baquie M, Abramia G (2008). Involvement of fumarase C and NADH oxidase in metabolic adaptation of *Pseudomonas fluorescens* cells evoked by aluminum and gallium toxicity.. Appl Environ Microbiol.

[22] Lee JH, Yang ES, Park JW (2003). Inactivation of NADP+-dependent isocitrate dehydrogenase by peroxynitrite. Implications for cytotoxicity and alcohol-induced liver injury.. J Biol Chem.

[23] Schneider K, Kastner CN, Meyer M, Wessel M, Dimroth P (2002). Identification of a gene cluster in *Klebsiella pneumoniae* which includes citX, a gene required for biosynthesis of the citrate lyase prosthetic group.. J Bacteriol.

[24] Kanao T, Fukui T, Atomi H, Imanaka T (2001). ATP-citrate lyase from the green sulfur bacterium *Chlorobium limicola* is a heteromeric enzyme composed of two distinct gene products.. Eur J Biochem.

[25] Buch A, Archana G, Naresh Kumar G (2010). Heterologous expression of phosphoenolpyruvate carboxylase enhances the phosphate solubilizing ability of fluorescent pseudomonads by altering the glucose catabolism to improve biomass yield.. Bioresour Technol.

[26] Varela-Gomez M, Moreno-Sanchez R, Pardo JP, Perez-Montfort R (2004). Kinetic mechanism and metabolic role of pyruvate phosphate dikinase from *Entamoeba histolytica*.. J Biol Chem.

[27] Heinzelmann S, Bauer G (2010). Multiple protective functions of catalase against intercellular apoptosis-inducing ROS signaling of human tumor cells.. Biol Chem.

[28] Gebicka L, Didik J (2009). Catalytic scavenging of peroxynitrite by catalase.. J Inorg Biochem.

[29] Blancato VS, Repizo GD, Suarez CA, Magni C (2008). Transcriptional regulation of the citrate gene cluster of *Enterococcus faecalis* Involves the GntR family transcriptional activator CitO.. J Bacteriol.

[30] Borisov VB, Forte E, Giuffre A, Konstantinov A, Sarti P (2009). Reaction of nitric oxide with the oxidized di-heme and heme-copper oxygen-reducing centers of terminal oxidases: Different reaction pathways and end-products.. J Inorg Biochem.

[31] Denayer S, Matthijs S, Cornelis P (2006). Resistance to vanadium in *Pseudomonas fluorescens* ATCC 17400 caused by mutations in TCA cycle enzymes.. FEMS Microbiol Lett.

[32] Varma SD, Hegde KR (2007). Lens thiol depletion by peroxynitrite. Protective effect of pyruvate.. Mol Cell Biochem.

[33] Frenzel J, Richter J, Eschrich K (2005). Pyruvate protects glucose-deprived Muller cells from nitric oxide-induced oxidative stress by radical scavenging.. Glia.

[34] Das UN (2006). Is pyruvate an endogenous anti-inflammatory molecule?. Nutrition.

[35] Mailloux RJ, Puiseux-Dao S, Appanna VD (2009). Alpha-ketoglutarate abrogates the nuclear localization of HIF-1alpha in aluminum-exposed hepatocytes.. Biochimie.

